# Inference of gene pathways using mixture Bayesian networks

**DOI:** 10.1186/1752-0509-3-54

**Published:** 2009-05-19

**Authors:** Younhee Ko, ChengXiang Zhai, Sandra Rodriguez-Zas

**Affiliations:** 1 Department of Computer Science, University of Illinois at Urbana-Champaign, Urbana-Champaign, IL, 61801, USA; 2 Institute for Genomic Biology, University of Illinois at Urbana-Champaign, Urbana-Champaign, IL, 61801, USA; 3 Department of Animal Sciences, University of Illinois at Urbana-Champaign, Urbana-Champaign, IL, 61801, USA

## Abstract

**Background:**

Inference of gene networks typically relies on measurements across a wide range of conditions or treatments. Although one network structure is predicted, the relationship between genes could vary across conditions. A comprehensive approach to infer general and condition-dependent gene networks was evaluated. This approach integrated Bayesian network and Gaussian mixture models to describe continuous microarray gene expression measurements, and three gene networks were predicted.

**Results:**

The first reconstructions of a circadian rhythm pathway in honey bees and an adherens junction pathway in mouse embryos were obtained. In addition, general and condition-specific gene relationships, some unexpected, were detected in these two pathways and in a yeast cell-cycle pathway. The mixture Bayesian network approach identified all (honey bee circadian rhythm and mouse adherens junction pathways) or the vast majority (yeast cell-cycle pathway) of the gene relationships reported in empirical studies. Findings across the three pathways and data sets indicate that the mixture Bayesian network approach is well-suited to infer gene pathways based on microarray data. Furthermore, the interpretation of model estimates provided a broader understanding of the relationships between genes. The mixture models offered a comprehensive description of the relationships among genes in complex biological processes or across a wide range of conditions. The mixture parameter estimates and corresponding odds that the gene network inferred for a sample pertained to each mixture component allowed the uncovering of both general and condition-dependent gene relationships and patterns of expression.

**Conclusion:**

This study demonstrated the two main benefits of learning gene pathways using mixture Bayesian networks. First, the identification of the optimal number of mixture components supported by the data offered a robust approach to infer gene relationships and estimate gene expression profiles. Second, the classification of conditions and observations into groups that support particular mixture components helped to uncover both gene relationships that are unique or common across conditions. Results from the application of mixture Bayesian networks substantially augmented the understanding of gene networks and demonstrated the added-value of this methodology to infer gene networks.

## Background

Relationships between genes and gene pathways can be inferred based on gene expression profiles across conditions, treatments, or samples obtained from microarray experiments [1-4]. Results from these studies can aid in the confirmation of previously known pathways and motivate the study of newly uncovered relationships among genes. A Bayesian network approach is well-suited to detect relationships between genes using an acyclic direct graph [1,2,4]. Needham et al. provided an in depth primer on learning Bayesian networks for computational biology [5]. This approach has a solid theoretical foundation, offers a probabilistic framework to describe the variation typically observed in microarray data, accommodates missing data, and incorporates prior knowledge on gene relationships.

Several implementations of Bayesian networks to infer gene networks have been reported [1-4,6,7] and two potential weaknesses have been identified. First, some applications require the transformation of continuous gene expression data into discrete input data, and this can influence the resulting network or resulting in loss of information. Thus, analysis of data that has not been discretized is favored. Second, most Bayesian network implementations assume standard binomial, multinomial probability or single Gaussian probability density functions of gene expression across a wide range of conditions. These distributions may fail to accommodate multimodal or skewed distributions associated with condition-dependent networks that exhibit changes in gene expression or gene relationships across conditions. This is because gene network inference is typically based on gene expression measurements across a wide range of conditions. Mixture models can be used to address this limitation because these models can describe potentially complex distributions of gene expression across a wide range of conditions. Newman and Leicht explored the capability of probabilistic mixture models to detect a very broad range of network structures without prior knowledge [8]. Ko et al. presented preliminary results from the application of Bayesian mixture algorithm to infer pathways [9]. They presented a histone pathway, did not use parameter estimates to identify condition-dependent gene relationships, and did not use cross-validation to assess the adequacy of the inferred network.

In this study, gene networks were inferred using a mixture Bayesian network approach. The superiority of this approach was evidenced on the new insights into general and condition-dependent relationships between genes and gene expression profiles gained while addressing some of the limitations encountered in previous applications of Bayesian network to infer gene networks. We demonstrate that the integration of mixture models and Bayesian network is well-suited to infer the structure of gene networks from continuous gene expression measurements across a wide range of conditions. The estimated parameters were easy to interpret, and aided in the characterization of gene expression and co-expression profiles. Furthermore, functions of these parameter estimates allowed the identification of condition-dependent networks. The mixture Bayesian network approach was used to infer three pathways on three independent data sets. The first prediction of the circadian rhythm pathway in honey bees and the first prediction of a cell communication pathway (adherens junction) in mouse embryos were obtained. In addition, the mixture Bayesian network approach was applied to a yeast cell-cycle data set commonly used in many gene network studies.

Comparisons of the predicted and known pathways and benchmark tests confirmed the outstanding performance of the mixture Bayesian network approach. Novel insights into general and condition-specific gene relationships and expression patterns were obtained, thus demonstrating the strength of the proposed approach to infer gene networks.

## Methods

### Mixture Bayesian Network Model

Bayesian networks can be described as a directed acyclic graph with nodes representing random variables or genes and directed edges representing the relationships between the nodes [5,10]. Given a set of genes {g_1_, g_2_,..., g_N_}, the corresponding Bayesian network *G *is represented as the joint probability distribution over all genes in *G *or *P(G)*. Applying the narrow sense form of the Markov property for a stochastic process, each gene is independent of non-descendant genes in the network, given the parent genes or nodes. This conditional independence property allows the factorization of the joint probability distribution as the product of conditional probabilities. The overall network is conceived as a set of gene subnetworks, each corresponding to a given gene node *g*_*j *_and its associated parent gene nodes *a*(*g*_*j*_) for *j *= 1 to *N*.

Models that accommodate for potential changes in gene expression and co-expression patterns across conditions are necessary when gene expression data across multiple conditions is used to infer gene networks. Mixture models are a flexible and effective option to describe changes in gene co-expression patterns across conditions because the joint probability functions of sub-networks are modeled with a combination of mixture components. The concept of integrating mixtures of Gaussian densities into Bayesian networks was introduced by Davies and Moore [11], and applied to word and social networks by Newman and Leicht [8]. Under the Bayesian network framework,



where *N *is the total number of genes (*j *= 1 to *N*) in the network (or total number of sub-networks because each gene specifies a sub-network) and *a*(*g*_*j*_) is the set of parent genes of child gene *g*_*j *_in the *j-th *sub-network. The conditional probability density function of the *j-th *gene *P*(*g*_*j *_| *a*(*g*_*j*_)), given the set of parent genes is expressed as the ratio between the joint probability density function of the parent and child genes in the *j-th *sub-network (*P*(*g*_*j*_, *a*(*g*_*j*_))), and the marginal probability density function of the parent genes in the *j-th *sub-network (*P*(*a*(*g*_*j*_))).

The joint and marginal probability density functions are assumed to follow a multivariate Gaussian distribution and a mixture model is used to describe possible sub-network structures. The probability density function (*P*) for the *j-th *sub-network is represented with a mixture of *K*_*j *_multivariate Gaussian distributions (*f*_*jk*_) each with a weight *α*_*jk*_. Here, **x**_*ji *_denotes a *p*_*j *_dimensional vector including the *i-th *expression observation (*i *= 1 to *D *where *D *is the total number of gene expression observations) of the child and parent genes in the *j-th *sub-network. For example, if *g*_*j *_has three parents then *p*_*j *_= 4. Therefore, the joint probability density function for the *j-th *sub-network is



and



where . For the joint probability density function, each component *k *of the mixture is described with a mean vector ***μ***_*jk *_of dimension *p*_*j *_and a variance-covariance matrix Σ_*jk *_of dimension *p*_*j *_× *p*_*j*_. The marginal probability density function of the (*p*_*j *_- 1) parent genes *a*(*g*_*j*_) is described in the same way as the joint probability density function but the dimensions of the *i-th *vector of observations , the mean vector  and the variance-covariance matrix  are (*p*_*j *_- 1), (*p*_*j *_- 1), and (*p*_*j *_- 1) × (*p*_*j *_- 1), respectively.

Therefore, the overall likelihood of the data across all genes in the network *G *is:



An example of a Bayesian gene sub-network is provided in Additional file 1. The relationship between the child and parent genes in each sub-network can be described by the weighted sum of the correlations between the parent geneand child gene estimated for each mixture component. The weights correspond to the mixture component weights. This computation was described by Bland and Altman [12] and is presented in Additional file 1.

### Overall Network Learning Algorithm

The learning algorithm integrated two major components, a) the inference of each individual gene sub-network structure and b) the inference of the overall network structure. The first component consisted of the identification of the sub-network for each gene (i.e. the parent genes of each child gene), the estimation of the parameters of the mixture models using the Expectation-Maximization algorithm [13,14], and the identification of the number of components of the mixture model best supported by the data. The second component consisted of the combination of the individual gene sub-networks into an overall network (exploiting the conditional independence of the gene nodes given the parent gene nodes in the Bayesian network framework), and the removal of cyclic relationships between genes in the overall network.

A detailed description of the network structure learning approach is presented in Additional file 2. The equations in the Expectation-Maximization steps corresponding to a gene sub-network follow the derivations of Bilmes [15] and are summarized in Additional file 3. The Bayesian Information Criterion (BIC) was used to identify the mixture model best supported by the data (i.e. optimal number of mixture components and associated parameter estimates) for each gene sub-network, and to evaluate the overall network [16]. Bayesian Information Criterion offers a good compromise between model adequacy and parsimony, minimizing the possibility of over-fitting the data with highly parameterized networks. This feature of the BIC is advantageous in gene network inference because of the large number of relationships and conditions that could potentially be considered.

Newman and Leicht used probabilistic mixture models to infer a wide range of network structures and noted that the convergence of the algorithm to the global maxima is not guaranteed [8]. To address this potential problem, we followed the approach implemented by Newman and Leicht, and networks were inferred using a range of starting points for each mixture parameter [8]. Each network presented in this study had the highest likelihood over all sets of initial conditions or runs evaluated. Furthermore, Davies and Moore demonstrated (based on ten-fold cross-validation) the suitability of mixtures of Gaussian densities estimated with the EM algorithm to infer Bayesian networks using data from an extensive astronomical survey and a high-throughput biological cell assay [11]. Extensive evaluation of the performance of the mixture Bayesian network approach to infer networks was presented by Newman and Leicht and Davies and Moore [8,11]. In this study, we concentrated on the application of this methodology to infer gene networks using microarray data, and demonstrated how the interpretation of parameter estimates and function thereof offered new insights into the relationships between genes.

A unique advantage of the integration of the mixture models and Bayesian network is the ability to uncover general and condition-specific gene networks. The parameter estimates of each mixture component of a gene sub-network were used as indicators of the gene expression level (mean or ***μ***), variation (variance or diagonals of **Σ**), and co-variation between genes (co-variance or off-diagonals of **Σ**). In addition, the mixture parameter estimates were used to compute the odds that the observation corresponded to a particular mixture component relative to the other components. Each sample had a non-zero probability (*albeit *small in some mixtures and networks) to pertain to each of the mixture components. For ease of interpretation and to overcome the limitation of using a single probability threshold across sub-networks with different number of mixture components, no threshold was used to assign samples to mixtures. Instead, the samples were assigned to the mixture component that had the highest odds. The assignment of samples to the most likely mixture component allowed the identification of samples (i.e. conditions or treatments) that shared the same expression and co-expression patterns for a gene sub-network.

### Data

Gene networks corresponding to three gene pathways (circadian rhythm, adherens junction, and cell cycle) were inferred from three microarray gene expression data sets (honey bee maturation, mouse embryo development, and yeast synchronization) using a mixture Bayesian network approach. The goal of these applications was to gain insights into general and condition-dependent relationships between genes and gene expression profiles, and demonstrate the flexibility and adequacy of the mixture Bayesian network approach to characterize networks.

#### Honey bee data set and circadian rhythm pathway

A detailed description of the honey bee microarray data set is presented by Whitfield et al. and Rodriguez-Zas et al. [17,18]. Briefly, gene expressions during behavioral maturation in the brains of honey bees (*Apis mellifera *or *A. mellifera*) from two subspecies (*A. m. mellifera *and *A. m. ligustica*) rose in one of two host colonies (*A. m. mellifera *and *A. m. ligustica*) representing two different environments were measured. Within combinations of bee and host race (*A. m. mellifera – A. m. mellifera *or MM, *A. m. mellifera – A. m. ligustica *or ML, *A. m. ligustica – A. m. mellifera *or LM, and *A. m. ligustica – A. m. ligustica *or LL), nurse bees were sampled on days 0, 4, 8, 12 and 17 after adult emergence (denoted h0, h1, h2, h3, and h4, respectively) and forager bees (denoted f4) were sampled at day 17 after emergence. The experimental design allowed the division of the honey bee data set into four independent bee-host subspecies-combination sub-data set (MM, ML, LM, and LL). Thus, the gene network was inferred on the complete data set and also on each of the four sub-data set. Processing of the data from 108 spotted cDNA microarrays included background subtraction and log2-transformation of fluorescence intensities, and removal of flagged spots or spots that did not surpass a minimum intensity threshold of 200. The log2 intensity values were normalized using a loess transformation, and global dye and microarray effects were removed using the approach implemented Beehive . Six genes assigned to the fruit fly circadian rhythm pathway in the KEGG database  were present on the microarray platform. The KEGG identifier for the circadian rhythm pathway in the fruit fly is dme04710 (accessed on August 2008).

#### Mouse embryo data set and adherens junction pathway

Data from nine microarray experiments that evaluated the effect of multiple toxic agents on mouse (*Mus musculu*s) embryo gene expression levels were used to reconstruct the adherens junction pathway. A detailed description of this data set is provided by Rodriguez-Zas et al. [19]. Briefly, the experimental design consisted of two types of direct comparisons with reverse labeling; comparisons between samples that received different treatments (e.g. ethanol, methylmercury, low oxygen, metabolic toxin 2-chloro analogue of 2'-deoxyadenosine administered at different time points or doses), and comparisons between treated and control samples. A total of 90 microarrays from the same platform were available for analysis, and data processing and normalization followed the same protocols applied to the honey bee data set [19] and implemented in Beehive. Seven genes assigned to the mouse adherens junction pathway in the KEGG database were present on the microarray platform. The KEGG identifier for the adherens junction pathway in the mouse is mmu04520 (accessed on August 2008).

#### Yeast synchronization data set and cell cycle pathway

The mixture Bayesian network approach was used to predict the yeast (*Saccharomyces cerevisiae*) cell-cycle network using gene expression data reported by [20]. The data set consisted of gene expression measurements from 76 microarrays across six experiments. The yeast experiments evaluated yeast cell synchronization via the arrest of cells by one of four conditions, followed by activation or release of the cells from the arresting condition. The synchronization conditions were cdc15-, cdc28-, *α *factor- and size or elutrition- based synchronization, and samples were obtained at various time points after the removal of the last four arresting conditions. In a separate study, the effects of inducing either the G1 cyclin Cln3p (Cln3) or the B-type cyclin Clb2p (Clb2) were examined, each in two experiments. The normalized gene expression data is available at the Stanford Microarray Database  and the experiment identifiers are: 9837, 9838, 1666, 1674, 1679, 49 to 58, 634 and 2141. A network including 14 genes analyzed by Friedman et al. using multinomial and single Gaussian distribution was inferred and results were compared [2]. The KEGG identifier for the yeast cell cycle pathway is sce04111 (accessed on August 2008).

#### Validation of the inferred gene networks

The relationships between genes inferred by the mixture Bayesian network approach were compared to relationships reported in the KEGG database, BioGRID  database, and Saccharomyces Genome database, or SGD , and in the literatures. These databases were accessed on August 2008 and provide complementary information. The KEGG database includes cause-effect or directional and non-directional relationships between genes depicted in pathway diagrams. In directional relationships, the child and parent genes are identified, meanwhile in non-directional relationships, genes are linked but there is neither a parent (i.e. cause) nor a child (i.e. effect) gene. The BioGRID database lists all known interactions or undirected relationships between genes. Due to differences in the curation and annotation protocols of each database, BioGRID can encompass more relationships than KEGG, but these relationships do not have associated direction.

Two strategies were used to confirm the reliability of the gene networks predicted by the mixture Bayesian network approach. First, a k-fold cross-validation approach was implemented. The design of the circadian rhythm experiment allowed to infer the gene network in each of four independent data sets corresponding to the four bee-host race combinations that encompassed the same five behavioral maturation stages or ages. Assessment of the mixture Bayesian network approach consisted on the comparison of the four inferred networks. Second, a permutation-based resampling approach was used to generate 400 randomized data sets with the same structure as the original circadian rhythm data set. These data sets were analyzed using the mixture Bayesian network approach and the predicted networks were compared to the observed network and the known pathway in the database.

Not all genes present in the pathway databases had reporters in the microarray platforms studied and thus two types of gene relationships, direct and indirect (not to be confused with directional and non-directional) were inferred. Direct relationships were detected between gene nodes that were present in the microarray platform and directly linked on the pathway database used to evaluate the results (e.g. KEGG). Indirect relationships were detected when the database indicated relationships between genes that were related through intermediate genes not present on the microarray platform.

The adequacy of the approach was demonstrated in the three independent data sets using a bilateral strategy. First, our approach encompasses a wide range of network models, from no relationships between genes and single Gaussian distributions to networks with variable number of relationships across sub-networks and mixtures of multiple Gaussian distributions. Our approach identified the gene relationships and number of mixture components best supported by the data for each sub-network, accounting for model complexity and data available using the BIC score. Multiple mixture components were favored over single components in the vast majority of the sub-networks inferred in the three data sets studied suggesting that adequacy of our approach to describe gene networks. Second, the inferred networks were compared to known network and the vast majority of the relationships were confirmed.

## Results and discussion

### Honey bee data set and circadian rhythm pathway

**Figure 1:**
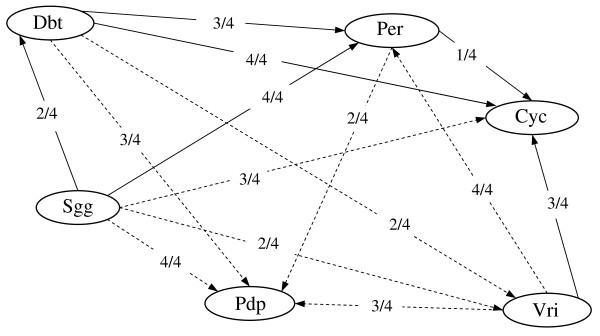
**Comparison of the predicted and expected relationships between genes pertaining to the circadian rhythm pathway based on four honey bee microarray sub-data sets**. Solid arrows (edges) denote direct relationships predicted and confirmed in the fruit fly KEGG pathway. Dashed arrows represent indirect relationships predicted and confirmed in the fruit fly KEGG pathway. The number besides each edge denotes the fraction of sub-data sets that supported the edge. Genes: *Per *= Period clock; *Cyc *= Cycle; *Vri *= Vrille; *Pdp *= Pyruvate dehydrogenase phosphatase 1; *Sgg *= Shaggy; *Dbt *= double-time.

The mixture Bayesian network approach provided the first published description of the relationship between genes in the circadian rhythm pathway of honey bees. In addition, this approach offered novel information on general and condition-specific gene relationships. Figure 1 summarizes the circadian rhythm gene network predicted by the mixture Bayesian network approach on each of the four bee-host sub-data sets used for cross-validation. Solid and dash-dotted arrows (edges) denote direct and indirect relationships, respectively, confirmed in the circadian rhythm pathway for the fruit fly in the KEGG database. The number beside each edge denotes the fraction of data sets that supports that edge. For example, 4/4 denotes that the gene relationship was detected on all four sub-data sets.

All direct and indirect relationships detected by the mixture Bayesian network on the honey bee genes were consistent with the relationships depicted for the fruit fly circadian rhythm pathway on the KEGG database. In addition, there were no predicted relationships that were not present on the KEGG fruit fly pathway. Previous reports indicated that maturation age was the highest source of differentially expressed genes in the honey bee microarray study under consideration, followed by the subspecies of the bee sampled and the species of the host colony [17,18]. In agreement with these studies, the mixture Bayesian network consistently detected most edges of the circadian rhythm pathway across multiple bee-host sub-data sets (edge ratios 4/4 or 3/4). However, not all relationships were detected in all four sub-data sets.

Four edges were only detected on half of the sub-data sets (edge ratio 2/4), and one edge was only detected on one sub-data set (edge ratio 1/4). The inability to consistently predict the same edges on all four sub-data sets may be due to two reasons. First, previous work noted that the subspecies of the honey bee sampled was another source of differentially expressed genes, *albeit *less important than maturation [17,18]. Differences on edge prediction among sub-data sets may be due to differences in the honey bee subspecies used in each sub-data set. Second, the size of each bee-host sub-data set may have resulted in insufficient information to predict some edges in some sub-data sets. To test this hypothesis, the mixture Bayesian network approach was applied to the complete data set. All the edges depicted in Figure 1 were also predicted on the complete data set. This result suggests that most or all data sets contained consistent information on the edges, although the information within data set may have not permitted an accurate prediction of some edges. Alternatively, these gene relationships were present on one or two sub-data sets, but the strength of the relationship on these sub-data sets was sufficient to be detected across all four sub-data sets. All the edges in Figure 1 were also inferred when the complete honey bee data set was analyzed and thus, results based on the complete honey bee data set are discussed.

The reliability of the gene network predicted by the mixture Bayesian network approach was assessed using permutation-based resampling. Of the networks predicted from the randomized data sets, 15% and 24% did not share any or only shared one relationship with the network inferred from the original data and confirmed against the KEGG database, respectively. The maximum number of gene relationships in common between the randomized and original data sets was five. Only 1% and 7% of the networks predicted from the randomized data sets shared five and four relationships out of the 14 gene relationships with the network originally inferred, respectively. Similarly, 32% of the networks predicted from randomized data sets had more than eight false positive relationships and only 5% of networks had four or less false positives relationships. The hegemony of these findings further advocates the capability of the mixture Bayesian network approach to uncover evidence on gene relationships contained in the data and minimize the probability of networks predicted by chance alone.

The insights into condition-dependent gene relationships offered by the mixture Bayesian network were evident on the sub-network of gene *Cyc*, including parent genes *Pe*r, *Dbt*, and *Vri*. Using the complete honey bee data set, the *Cyc *sub-network was best described by a mixture of two components. The parameter estimates corresponding to each mixture component of a gene sub-network offered information on the expression and co-expression patterns. Mixture components 1 and 2 had weights equal to 0.35 and 0.65, respectively. The most notable differences between the two mixture components were the level of gene *Cyc *(double in mixture component 2 relative to mixture component 1) and the correlations between gene *Cyc *and parent genes *Vri *and *Sgg*. The correlations of *Cyc *with both parent genes were negative in mixture component 1 and positive in mixture component 2 and the difference between the correlations of both mixture components was significant at P-value < 0.04 and < 1.2 × 10^-10 ^for *Cyc *and *Vri *and *Cyc *and *Sgg*, respectively [21].

Table 1 summarizes the assignment of samples from six maturation ages (h0, h1, h2, h3, h4, and f4) and four bee-host combinations (LL, LM, ML, and MM) to each of the two mixture components (Mix1 and Mix2) for the *Cyc *gene sub-network. Due to the experimental design, the number of samples differed across bee-host-age combinations [17]. The *Cyc *sub-network was better described by mixture component one (Mix1) for most *A. m. ligustica *bees raised on *A. m. ligustica *hives (LL), meanwhile mixture component two (Mix2) better described the sub-network for *A. m. mellifera *bees raised on *A. m. mellifera *hives (MM). Their results are consistent with differences in expression across bee subspecies reported by Whitfield et al. [17]. In addition, the mixture component one description of the *Cyc *sub-network was preferred for one-day old nurse (h0) and forager (f4) bees, meanwhile mixture component two was favored for four, eight, and twelve-day old nurse (h1, h2, and h3, respectively) bees. This result is consistent with Whitfield et al. who concluded that many changes in gene expression across maturation ages occurred by day eight [17]. Rodriguez-Zas et al. also reported a major difference in the gene expression profiles of LL and LM bees at the eight and twelve-day maturation ages [18].

**Table 1:** Distribution of the observations pertaining to bee-host subspecies combinations and maturation age conditions based on a mixture model with two components for the Cyc gene sub-network.

Mixture	Condition					
	LL	LM	ML	MM		

Mix1	34	28	39	20		

Mix2	20	26	15	34		

Age:	h0	h1	h2	h3	h4	f4

Mix1	32	20	8	14	12	35

Mix2	4	28	16	34	12	1

Bee-host subspecies combinations: LL, LM, ML, and MM.

Maturation ages: h0, h1, h2, h3, h4, f4

Mix1 and Mix2: mixture components 1 and 2, respectively.

The mixture Bayesian network approach was able to identify different models (or mixture components) of association between genes and of gene expression profiles depending on the conditions under consideration, and also provided a general model that is the weighted average of the condition-specific components. Cyran et al. noted that the fruit fly circadian clock encompasses two interlocked transcriptional feedback loops [22]. In one loop, *dClock/Cyc *activates the expression of *Per*, and the *Per *protein in turn inhibits the activity of *dClock/Cyc*. In addition, *Vri *and *Pdp1 *encode related transcription factors whose expressions are directly activated by *dClock/Cyc*. In the second loop, the *Vri *and *Pdp1 *proteins feed back and directly regulate the expression of *dClock*. Results from the mixture Bayesian network approach (Figure 1 and Table 1) confirm these findings.

The identification and interpretation of general and condition-dependent gene co-regulation patterns, using the odds that observations pertain to each mixture component, and the mixture component parameter estimates presented for the gene *Cyc *sub-network can be repeated for all other genes in the network. Although the optimal number of mixture components varied across gene sub-networks (in most cases a mixture of two or three components was estimated), similar general and condition-dependent conclusions were obtained for other gene sub-networks in the circadian rhythm pathway and thus are not presented here. This situation may be due to the high degree of interconnectedness among the gene nodes in this network. For networks encompassing less related sub-networks, parameter estimates from each sub-network module should be evaluated.

Rubin et al. postulated, based on phylogenetic and correlation analyses of the function and domains of two proteins (*Cry *and *Tim*), that the circadian rhythm pathway of the honey bees resembles more the mammalian than the fruit fly counterpart [23]. The similarity between the honey bee and fruit fly circadian rhythm networks found in the present study may be due to absence of reporters associated with genes that differ across insect species, such as the genes considered by Rubin et al., on the microarray study under consideration [23].

Several indirect relationships among genes reported in the fruit fly KEGG pathway were predicted as direct ones due to the absence of microarray reporters corresponding to the intermediate genes. Although the identification of relationships (direct or indirect) among genes constitutes a major step towards a comprehensive description of biological pathways, precise characterization of the relationships is also important. Differences between the predicted and reported (e.g. literature, databases) gene relationships may be due to differences among the experiments considered (e.g. honey bee *versus *fruit fly) and insufficient or inaccurate information (e.g. small experiment, limited microarray platform). Additional studies are necessary to ascertain the true nature (direct or indirect) of these gene relationships.

### Mouse embryo data set and adherens junction pathway

**Figure 2:**
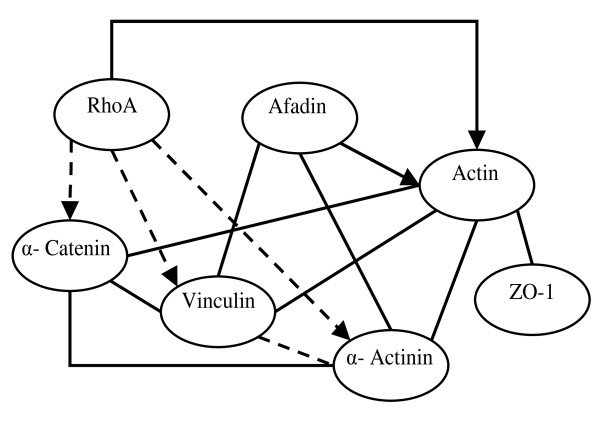
**Comparison of the predicted and expected relationships between genes pertaining to the adherens junction pathway based on mouse embryo microarray data**. Solid lines and arrows (edges) denote direct relationships predicted and confirmed in the mouse KEGG pathway. Dashed lines and arrows represent indirect relationships predicted and confirmed in the KEGG pathway. Arrows denote directional relationships reported in the KEGG pathway. Lines denote non-directional relationships reported in the KEGG pathway. Genes: *RhoA *= ras homolog gene family, member A; *Afadin *= mixed lineage-leukemia translocation to 4 homolog; *Actin *= Actb; *α-Catenin *= Ctnna1; *Zo-1 *= tight junction protein 1; *α-Actinin *= Actn1; *Vinculin *= Vcl.

Yokoyama et al. studied the relationship among various proteins in the adherens junction pathway [24]. Using the mixture Bayesian network approach, the first depiction of general and condition-specific gene profiles and interactions in the adherens junction pathway of mouse embryos was obtained. Figure 2 depicts the adherens junction gene network predicted by the mixture Bayesian network approach.

This approach predicted directional relationships in which the parent and child gene nodes are identified. However, the adherens-junction mouse pathway in the KEGG database included some non-directional gene relationships. Because the inferred network was validated against the KEGG pathway, non-directional gene relationships are denoted with lines instead of arrows. All direct and indirect relationships between genes predicted by the mixture Bayesian network approach were present in the KEGG database. Consistency between the predicted and reported adherens junction networks was also observed for two additional pathways (actin cytoskeleton and axon guidance) predicted using the same mouse microarray data set [19,25].

The parameter estimates and odds that observations pertained to different mixture model components enhanced the understanding of the differences in gene profiles and relationships between mouse embryo treatments. For example, a mixture of three components was best suited to describe the sub-network of gene *α-Actinin*. Mixture components one, two and three had weights 0.28, 0.57 and 0.16 and modeled low, intermediate and high levels of expression of all genes in the *α-Actinin *sub-network, respectively. In addition, gene *α-Actinin *had a negative correlation with parent gene *Actin *in the first mixture component meanwhile this correlation was positive in the other two mixture components. The statistical significance of the difference between correlations from mixture components one and two, and one and three were P-value < 8.6 × 10^-10 ^and P-value < 1.1 × 10^-11^, respectively meanwhile the correlations from mixture components two and three were non-significantly different (P-value > 0.25).

Table 2 summarizes the assignment of observations within conditions (mouse embryo studies or series and treatments) to each of the two components (Mix1 and Mix2) of a mixture model describing the gene *Actin *sub-network. All or the majority of the observations within a series (or study) were assigned to a single mixture component. For example, all the observations within series GSE 1068, 1070, 1075, 1076, and 1079 were assigned to one mixture component. In addition, all observations except one were assigned to the same mixture component in series GSE 1069 and 1077. Detailed description of the difference between conditions within study is provided in Rodriguez-Zas et al. [19]. Briefly, most studies evaluated different dosages, dosage timing, or combination of agents that influence embryo development. The results from the mixture Bayesian network approach suggested that most of the variations in conditions within study had a minor effect on the relationship between genes in the *Actin *sub-network relative to the effect of study. In general, for other gene subnetworks the distribution of the observation assignments to mixture components was in agreement with the results from the *Actin *sub-network. The conditions with the most extreme distribution of observations were consistently found across gene subnetworks.

**Table 2:** Distribution of conditions pertaining to nine studies and associated treatments based on a mixture of two components for the *Actin *gene sub-network.

	Condition								
Series:	68	69	70	72	74	75	76	77	79

Mix1	5	1	4	2	4	5	7	1	6

Mix2	0	3	0	5	9	0	0	6	0

Treatment:	cnt	mhg	CdA	eth	Ro	PK	Oxg		

Mix1	3	11	10	2	3	5	5		

Mix2	7	7	3	8	4	6	0		

Studies: Gene Expression Omnibus series 1068, 1069, 1070, 1072, 1074, 1075, 1076, 1077, 1079.

Treatments: control or cnt, methylmercury or mhg, ethanol or eth, 2-chloro-2'deoxyadenosine or CdA, oxygen or oxg, PK11195 or PK, and Ro5-4864 or Ro. Mix1 and Mix2: mixture components 1 and 2 respectively.

Genes in sub-network: *Actin, RhoA, Afadin*, *α-Catenin*, *Vinculin*, *α-Actinin*, and *ZO-1*.

Consideration of the assignment of treatment observations, regardless of study, to the mixture components for the *Actin *sub-network revealed new and confirmed known biological interpretations (Table 2). For example, the only three conditions receiving 2-chloro-2'deoxyadenosine or CdA that were assigned to the mixture component Mix2 correspond to sampling intervals before (three hours) and after (six hours) the critical event of p53 protein induction expressed in cda-treated embryos between 3.0- and 4.5 h post-exposure (GSE 1069) and to embryos also treated with Ro5-4864 (denoted Ro), a presumed agonist that is weakly teratogenic (GSE 1072). All the observations during the critical event (4.5 hours) were assigned to Mix1. These results suggested that sampling time and treatments in addition to cda could have substantial impact on the *Actin *sub-network.

The odds that observations pertain to each mixture component also provided insights into the effects of two pharmacologic agents, PK11195 (denoted PK) and Ro5-4864 (Table 2). The PK agent can rescue embryos from the effects of toxic agents meanwhile Ro is a presumed agonist that can have weak teratogenic effects. The fairly even assignment of PK and Ro treatments to both mixture components suggested that the impact of these treatments on the *Actin *sub-network was dependent on the other conditions of the experiments. Similarly, the evaluation of the assignment of control (or untreated) samples to mixture components offered information on the behavior of seemingly similar samples. Out of ten control samples (cnt), seven were better described using Mix2. Among the three control samples assigned to Mix1, one corresponded to the mouse sub-strain C57BL/6J (B6J) and was consistent with the assignment of all other B6J samples that received treatment to Mix1. This finding suggested the potentially high effect of the genotype of the embryo on the relationship among genes in the *Actin *sub-network. The other two control samples assigned to Mix1 corresponded to series GSE 1069 and 1072, and these assignments were consistent with the assignment of all (GSE 1069) or the vast majority (GSE 1072) of the samples to Mix1. These assignments are consistent with the strong series effect previously discussed.

### Yeast synchronization data set and cell cycle pathway

**Figure 3:**
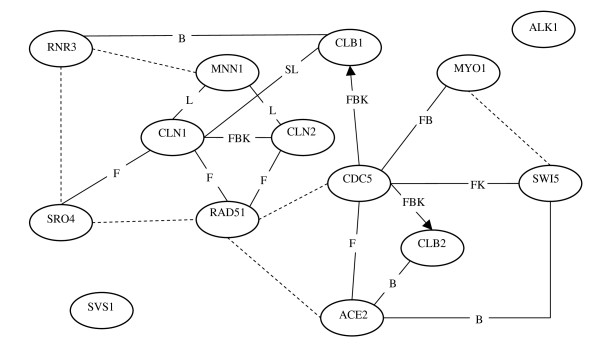
**Comparison of the predicted and expected relationships between genes in the yeast cell-cycle pathway based on yeast microarray data**. Solid lines and arrows denote direct relationships predicted and confirmed in the BioGRID (B), SGD (S), KEGG (K) databases, literature (L) or by Friedman et al. (F). Dashed lines denote indirect relationships predicted and confirmed in the databases. Arrows denote directional relationships reported in the KEGG pathway. Lines denote non-directional relationships reported in the KEGG pathway. Genes: *CLN1 *= Cln1p/G1 cyclin; *CLN2 *= Cln2p/G1 cyclin; *SRO4 *= *AXL2 *= Axl2p/Integral plasma membrane protein coding gene; *RAD51 *= MUT5; *MNN1 *= *α*-1,3-mannosyltransferase; *SWI5 *= *α*-1,3-mannosyltransferase; *SVS1 *= Cell wall and vacuolar protein coding gene; *ALK1 *= Alk1p/Protein kinase; *CLB1 *= B-type cyclin; *ACE2 *= Ace2p/transcription factor; *CDC5 *= Cdc5p/Polo-like kinase; *MYO1 *= Myo1p/Type II myosin heavy chain; *CLB2 *= B-type cyclin; *RNR3 *= ribonucleotide-diphosphate reductase.

Figure 3 summarizes the relationships between genes in the yeast cell-cycle pathway predicted by the mixture Bayesian network approach. The predictions were compared to directional and non-directional relationships depicted in the KEGG yeast cell-cycle pathway, listed in the BioGRID database and SGD, and predicted by Friedman et al. [2]. The predicted gene relationships with known direction are denoted with arrows. The only two directional gene relationships reported in the KEGG database are CDC5 → CLB1 and CDC5 → CLB2. The remaining gene relationships could only be confirmed in databases that list undirected relationships (BioGRID, SGD) and thus these relationships are depicted with lines instead of arrows. In Figure 3, the direct relationships confirmed in BioGRID, Friedman et al., KEGG, literature and SGD are denoted with solid lines and labeled with the letters B, F, K, L and S, respectively, meanwhile the indirect relationships are denoted with dashed lines.

Of the 21 gene relationships predicted by the mixture Bayesian approach, nine were confirmed in Friedman et al., seven and three were listed in the BioGRID and KEGG databases respectively, and three and one were reported in the literature [26,27] and SGD database, respectively. Several direct relationsh ips predicted by the mixture Bayesian network approach were not reported in the BioGRID, KEGG, literature, SGD databases or in Friedman et al. [2]. Likewise, only three out of the 13 directed relationships and four out of the 14 genes in the pathway predicted by Friedman et al. are present in the KEGG pathway [2]. Although experimental studies are necessary to confirm or disprove these findings, additional gene relationships and literature review imply that some are likely to be true (direct or indirect) relationships. For example, through text mining of biomedical literature, Liu et al. found that genes *MNN1*, *CLN1*, and *CLN2 *were all influenced by the same activators [28]. This finding supports the novel relationships between *MNN1 *and either *CLN1 *or *CLN2 *detected by the mixture Bayesian network approach. Had microarray data on the common activators been available, the relationship between these genes could have been further resolved. Additional studies are necessary to determine if the unconfirmed relationships are true positives instead of false positives. The mixture Bayesian network approach was not able to detect relationships between genes *SVS1 *and *ALK1 *and the rest of the genes in the network. These results are consistent with the absence of evidence relating genes *SVS1 *and *ALK1 *in the literature or databases.

Assessment of the mixture model parameter estimates was used to gain insights into the gene sub-networks. For example, a mixture model with two components receiving equal weight was found to describe the sub-network of gene *CDC5 *depicted in Figure 3. The mean expression of the genes in mixture component one was between a half and a third of the mean expression of the genes in mixture component two. In addition, genes *CDC5 *and *CLB1 *had a positive correlation (0.36) in mixture component two, meanwhile the correlation was weaker and negative (-0.17) in mixture component one and these correlations were different at P-value < 0.001).

The assignment of the samples to the components of the mixture model describing gene sub-networks helped in the understanding of the changes in the network across the different treatments or conditions studied. For example, consideration of the probability that a sample pertained to either one of the two components of the mixture model used to describe the sub-network of gene *CDC5 *confirmed some expected assignments and suggested unexpected commonalities between samples. The assignment of elutrition samples collected every 30 minutes to mixture components provided a good example of a slow transition of the network behavior across time. The first six samples collected between 0 and 150 minutes were assigned to one mixture component and six samples obtained from 240 to 390 minutes were assigned to the other mixture component. Samples collected at intermediate time points (between 180 and 210 minutes) had a similar odds ratio to pertain to either mixture component. A discussion of the assignment of samples in the *α*-factor, cdc28 and cdc15 synchronization is provided in Additional file 4.

Overall, the mixture Bayesian network approach detected more confirmed relationships than the approach presented by Friedman et al. [2]. However, Friedman et al. detected five relationships (*CLN2*:*SVS1*, *CLN2*:*RNR3*, *SVS1*:*MNN1*, *CDC5*:*SVS1*, *CDC5*:*ALK1*) that were not predicted by the mixture Bayesian approach and were not present in the BioGRID database. The higher number of relationships predicted by the mixture Bayesian network approach relative to the Bayesian network implementations of Friedman et al. may be due to the flexibility of the mixture model in the mixture Bayesian network approach to accommodate fluctuations on the gene patterns and relationships across the multiple conditions evaluated [2].

### Single, fixed-number, and optimal mixture densities

The mixture Bayesian network approach to infer gene networks implemented in this study used the EM algorithm to obtain parameter estimates and the BIC score to identify the optimal number of mixture components supported by the data. This framework offered great flexibility to accommodate a wide range of models and probability density functions across gene sub-networks and within gene sub-network, across the conditions under study. A series of benchmarking tests were conducted to evaluate the benefits of the more flexible and data-driven mixture Bayesian network approach for different scenarios.

**Figure 4:**
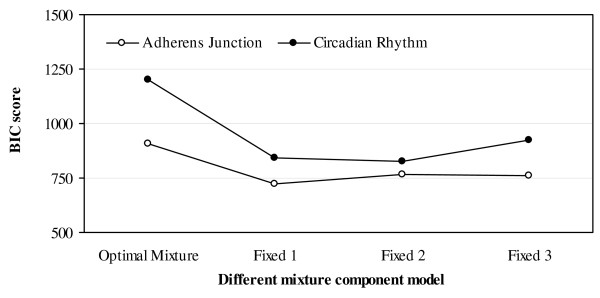
**The Bayesian information criterion (BIC) score of the predicted adherens junction and circadian rhythm networks based on honey bee and mouse embryo microarray data, respectively**. The number of mixtures is estimated from the data (Optimal Mixture) or fixed to one (Fixed1), two (Fixed2), or three (Fixed3) mixture components. Higher BIC score values indicate more adequate description of the data.

The description of the sub-networks supported by the data based on BIC score favored mixture over single distributions. This indicates that for the networks and data sets considered in our study, mixtures offer a better description of the relationship between genes than single distributions. This result was further validated when the assignment of samples from different treatments or conditions to mixture components was investigated. The robustness of the mixture Bayesian network learning process granted by the use of mixture distributions and potentially variable numbers of mixture components across the network was investigated. Figure 4 presents the BIC scores of the networks predicted using the mixture Bayesian network approach with the number of mixture components fixed to single or mixture of two or three Gaussian densities (Fixed1, Fixed2, and Fixed3 respectively), or estimated from the data (Optimal Mixture) for the circadian rhythm and adherens junction networks previously presented. The BIC score of the Optimal Mixture implementation was higher than the approaches that used a fixed number of mixtures across all subnetworks. The superiority of the mixture Bayesian network approach that estimates the optimal number of mixture components for each gene sub-network from the data over approaches with fixed number of components was evident in both networks. In addition, the maximum number of parents in a gene sub-network was set to five in this study, based on a review of pathways reported in the KEGG database and associated to the pathways studied. This maximum was supported by the fact that the optimal BIC score was always found with one to four parent genes.

### Computational evaluation

**Figure 5:**
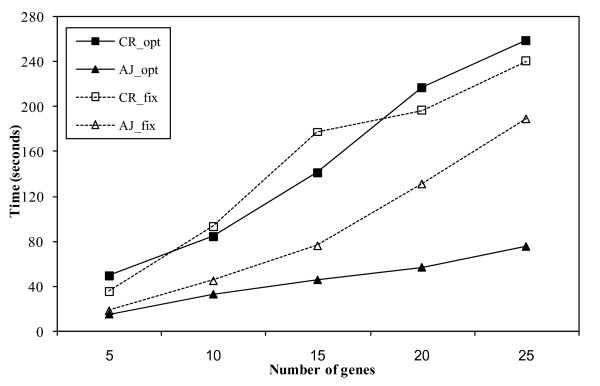
**Computational time (CPU in seconds) to predict networks of size 5 to 25 genes**. Values represent the average time across five data sets sampled at random from the circadian rhythm (CR) and adherens junction (AJ) microarray data sets per network size. The number of mixture components was estimated from the data (opt) or fixed at 3 components (fix).

**Figure 6:**
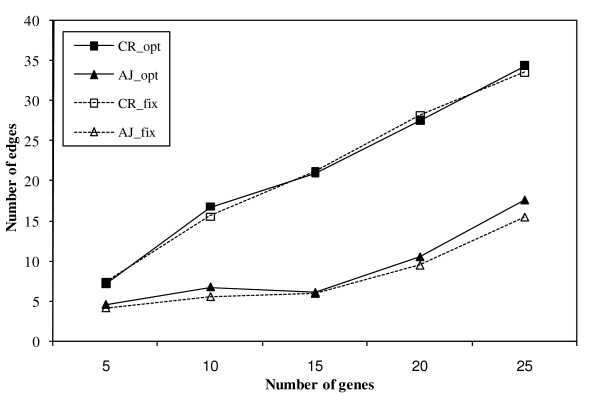
**Number of predicted relationships or edges in networks of size 5 to 25 genes**. Values represent the average number of edges across five data sets sampled at random from the circadian rhythm (CR) and adherens junction (AJ) microarray data sets per network size. The number of mixture components was estimated from the data (opt) or fixed at 3 components (fix).

The computational time required for the prediction of a network across a wide range of number of genes was evaluated. The mixture Bayesian network approach was implemented using the C++ programming language. Figure 5 shows the computational time (in CPU seconds using a AMD Opteron 248 2.2 GHz processor, after the microarray data was read) required to reconstruct networks of different size, ranging from five to 25 genes sampled at random from the circadian rhythm (CR) and adherens junction (AJ) microarray data sets. Five data sets were obtained from the complete microarray data sets for each network size considered, and a network was estimated for each data set and network size. The computing time to predict a network depends in part on the degree of connectivity between the genes studied. Because the gene sets were drawn at random from the microarray data sets, the degree of relationship between these genes (number of edges) was expected to vary across data sets within network size and across network sizes studied. Thus, both the number of edges predicted in each network and the computational time required to predict the network were recorded. Figure 5 and Figure 6 present the average computational time and number of edges across the five data sets within network size evaluated for two cases, when the number of mixture components was estimated from the data (opt) or fixed at 3 components (fix). These comparisons allowed the assessment of the impact of the mixture component of the algorithm on the computational time and inferred edges.

As expected, the higher the number of genes in the network, the higher the computational time required for estimation of the complete network (Figure 5). The lower computational time to infer the adherens junction network compared to the circadian rhythm network may be due to the fewer relationships (edges) detected in the first network (Figure 6). In the adherens junction network, the trends of computational time and number of edges across number of genes were similar. In the circadian rhythm network, the computing time increased at a slightly or moderately higher rate than the number of edges across the number of genes. The disparity between the trends of time and number of edges was higher in the adherens junction network with a fixed number of mixture components than when the number of components was estimated from the data. This result may be due to the potential inadequacy of the fixed number of mixture components to describe some or all gene sub-networks. Thus, more iterations and more time were required to estimate the model parameters and infer the network structure. Furthermore, the nearly constant number of edges (ranging from four to eight) inferred in the adherens junction across the number of gene nodes studied (ranging from five to 25) suggested that the mixture Bayesian network algorithm was not biased towards inferring higher number of edges with higher number of gene nodes network (Figure 6).

### Algorithmic extensions

The identification of a Bayesian network structure is an NP-hard problem because the number of possible candidate structures super-exponentially increases with the number of gene nodes. Two structure learning methods that have complementary advantages were evaluated in this study, the sparse candidate algorithm [1] and the simulated annealing method [29]. The sparse candidate algorithm achieves fast structure learning by restricting the search space of potential parent gene nodes. Intuitively we would like to restrict the search space as less as possible. In this study, the algorithm was modified to minimize the impact of restricting the search space, while also maintaining efficiency by evaluating additional parent genes for each gene sub-network with less than the maximum number of parent genes.

The network structure was also inferred using a simulated annealing algorithm to overcome the potential risk of inferring networks at local maxima instead of global maximum [30]. At each iteration, the simulated annealing algorithm randomly jumped from the current network topology to a nearby topology formed from the addition, deletion, or reversion of an edge in the current topology. Simulated annealing can escape from potential local minima by occasionally sampling from less suitable networks and the probability of jumping to another status is empirically adjusted. This procedure is repeated until convergence. The implementation of these two algorithms allowed the evaluation of the mixture Bayesian network approach to be independent of the actual algorithm used to learn the topology of the network. The vast majority of the gene relationships was predicted by both the sparse candidate and simulated annealing algorithms. Therefore, only results from the sparse candidate algorithm are presented here.

The mixture Bayesian approach presented here can be extended to include other gene (or variable) selection criteria, consider other distributional assumptions and prior information on the relationship between genes in a network. These extensions are discussed in Additional file 5.

## Conclusion

A comprehensive characterization of the circadian rhythm pathway in honey bees and of the adherens junction pathway in mouse embryos was obtained using a mixture Bayesian network approach. All the gene relationships detected by the mixture Bayesian network based on honey bee gene expression information were consistent with known relationships in the fruit fly circadian rhythm pathway. The mixture Bayesian network approach was also able to uncover changes in gene relationships and profiles in the *Cyc *gene sub-network associated with changes on the maturation age, subspecies of honey bee sampled, and subspecies of honey bees in the host hive. Likewise, all the gene relationships detected by the mixture Bayesian network using mouse-embryo gene expression information were consistent with known relationships in the general mouse adherens junction pathway. All or the vast majority of the samples within experiment were assigned to the same mixture component model in seven out of the nine experiments considered. This assignment of samples to mixture components suggested that experiment had a major impact on the gene relationships and expression profiles in the *Actin *gene sub-network. The application of the mixture Bayesian network approach to the well-studied yeast cell cycle confirmed many reported relationships. In addition, the assignment of samples to the components of the mixture model for the *CDC5 *gene sub-network provided novel insights into gene associations. For example, the assignment of elutrition samples to the mixture components suggested a slow transition of the network behavior across time. On the other hand, the assignment of cdc15-synchronized samples to mixture components suggested that the networks were instable across most of the time-points considered.

This study demonstrated that the mixture Bayesian network approach is well-suited to infer gene pathways based on microarray gene expression data. The estimation of the number of mixture components from the data allowed the simultaneous modeling of gene sub-networks with either constant or variable behavior across conditions. This modeling approach has two potential advantages, a) it can increase the accuracy of the inferred relationships and precision of the parameter estimates, and b) it can help identify groups or clusters of observations that support particular (condition-dependent) models or mixture components. The superior accuracy of the mixture Bayesian network approach was demonstrated by the better BIC score of the network with the number of mixture components estimated from the data, compared to the BIC score of approaches with a fixed number of mixture components. The mixture of Gaussian distributions demonstrated in this study naturally accommodated the continuous and typically normally-distributed gene expression measurements. The proposed approach can be extended to use mixtures of other discrete or continuous distributions, or incorporate prior information on the genes in the network.

The proposed mixture Bayesian network approach allows the detection of general and condition-specific relationships among genes, and the prediction of an overall network structure within the framework of probabilistic inference. The interpretation of parameter estimates and functions (e.g. probabilities and odds to pertain to a mixture component) can offer a more comprehensive understanding of gene patterns and relationships. Insights into the gene patterns, and the relationships between them, can be used to design effective follow-up experiments and investigate the novel relationships predicted by the mixture Bayesian network approach.

## Authors' contributions

YK implemented the mixture Bayesian network algorithm, inferred the three gene networks, contributed to the interpretation of results, and drafted the manuscript. C-XZ helped interpret the algorithm and reviewed the manuscript. SRZ obtained funding for the study, participated in its conception, coordination, interpretation of results, and helped write the manuscript. All authors have read and approved the final version of this manuscript.

## Supplementary Material

Additional file 1 **Representation of a mixture model for the Bayesian sub-network corresponding to child gene Gene_j _and parent genes: Gene_1_, Gene_2_,..., Gene_p-1_**. The figures represent the Bayesian sub-network of the jth gene with p-1 parent genes. Click here for file

Additional file 2 **Illustration of the network-structure learning process**. Diagram of the steps of the algorithm to infer the topology of the network. Click here for file

Additional file 3 **Estimation of mixture parameters**. Formulae to estimate the parameters using the Expectation-Maximization algorithm. Click here for file

Additional file 4 **Assignment of yeast cell cycle conditions to mixture components**. Distribution of yeast cell cycle treatments across the mixture components of the gene sub-network. Click here for file

Additional file 5 **Additional algorithmic extensions**. Potential modifications of the proposed algorithm are discussed. Click here for file
