# Supplementary material for: Inference of gene pathways using mixture Bayesian networks
Source: BMC Syst Biol. 2009 May 19;3:54. doi: 10.1186/1752-0509-3-54 (PMC2701418; doi:10.1186/1752-0509-3-54)
Supplement: Additional file 1 — Representation of a mixture model for the Bayesian sub-network corresponding to child gene Genej and parent genes: Gene1, Gene2,..., Genep-1. The figures represent the Bayesian sub-network of the jth gene with p-1 parent genes. [file 1752-0509-3-54-S1.pdf]

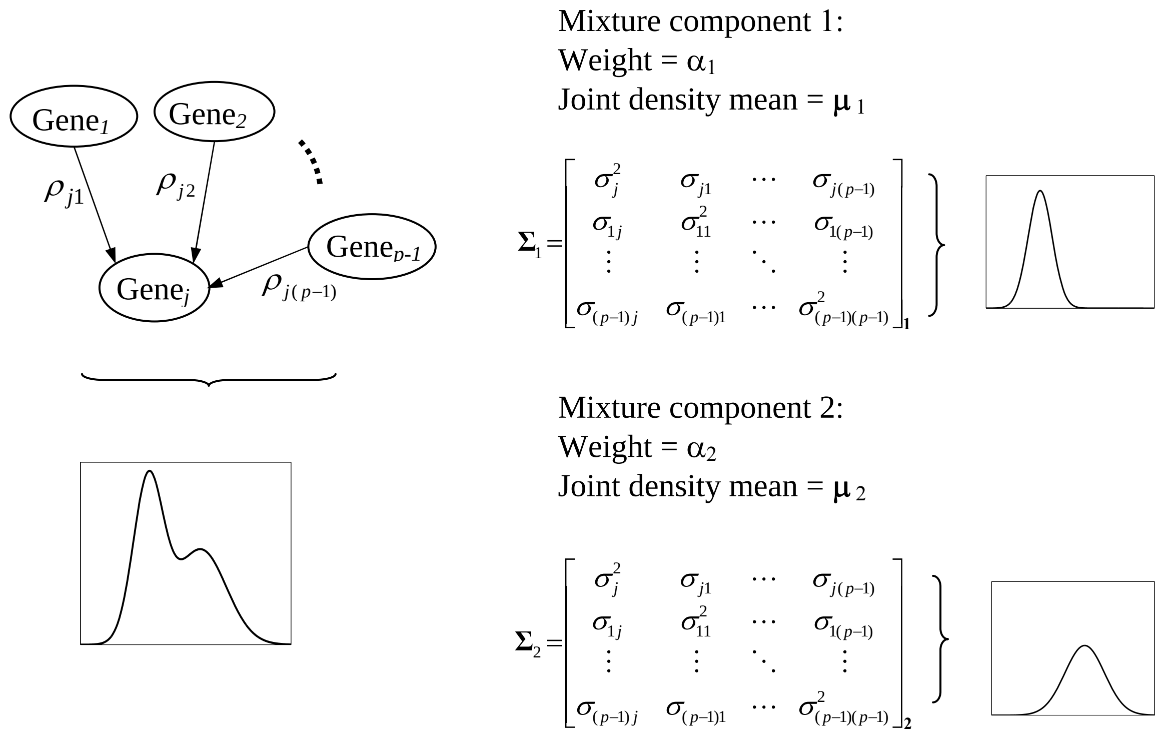

$$\rho_{jq} = \sum_{k=1}^{K_j} \alpha_k \left(\rho_{jq}\right)_k = \sum_{k=1}^{K_j} \alpha_k \left(\frac{\sigma_{jq}}{\sigma_j \sigma_q}\right)_k$$

The Bayesian gene sub-network example described in this Additional file corresponds to one gene node (*Gene*$_j$) with $(p - 1)$ parent genes (*Gene*$_1$, *Gene*$_2$, …, *Gene*$_{p-1}$). The joint probability density function for this sub-network composed of *Gene*$_j$ and parent genes is described with a mixture of two multivariate Gaussian distributions (k = 1, 2), each with a particular mean vector ($\boldsymbol{\mu}_k$) of dimension p and a variance-covariance matrix ($\boldsymbol{\Sigma}_k$) of dimension $p \times p$. The genes of a network may exhibit different expression and co-expression patterns across the conditions under study. This situation is likely to be better described by more than one density (e.g. Gaussian) combined into a mixture model where each component receives a weight, rather than a single density. For example, a gene (e.g. *Gene*$_2$) may have a strong influence on the expression of another gene (e.g. *Gene*$_j$) under certain conditions, and this strong association is better described by one mixture component. The influence of the former gene may be weak under another set of

conditions, and this weak association is better described by a different mixture component. The correlation ρ_{jq} between the $Gene_q$ $(Gene_q \in a(Gene_j))$ and $Gene_j$ within a mixture component is a function of the covariance and variance estimates that correspond to the off-diagonal and diagonal entries of Σ_k, weighted by the component weight α_k.
