# Supplementary material for: Inference of gene pathways using mixture Bayesian networks
Source: BMC Syst Biol. 2009 May 19;3:54. doi: 10.1186/1752-0509-3-54 (PMC2701418; doi:10.1186/1752-0509-3-54)
Supplement: Additional file 2 — Illustration of the network-structure learning process. Diagram of the steps of the algorithm to infer the topology of the network. [file 1752-0509-3-54-S2.pdf]

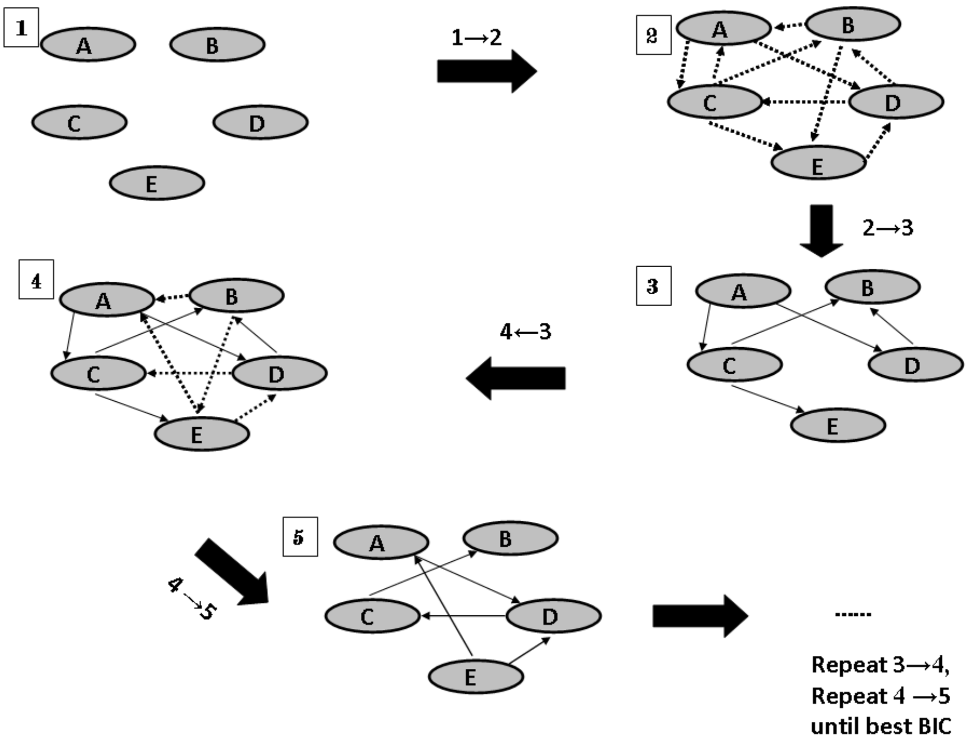

In this example, five gene nodes (A, B, C, D, and E) are denoted as ovals, edges or gene relationships within sub-network and overall network (after removal of cyclic relationships) are denoted with dotted and solid arrows, respectively. In this Additional file, Fig. 1 depicts the start of the algorithm, a overall network with all gene nodes disconnected. From 1→2, edges to all possible parent genes (up to a maximum number of parents) were evaluated for each child gene using the BIC score. The edges supported by the data are denoted with dotted lines, and all five child gene sub-networks are constructed. Fig. 2 depicts the integration of all gene sub-networks into a overall network that can include cyclic relationships, such as that between A and C. From 2→3, each cyclic relationship in the overall network was eliminated by removing the edge with the weakest BIC support. Fig. 3 depicts the overall network without cyclic relationships, and the remaining gene relationships are denoted with solid edges. The BIC score for the

overall network was computed. This network may accommodate additional edges, thus, from 3→4, additional edges were evaluated for each gene sub-network using the BIC score, and the edges supported by the data are denoted with dotted lines. The network in Fig. 4 (like Fig. 2) depicts the integration of all gene sub-networks into a overall network that can include cyclic relationships. In 4→5 (like from 2→3), cyclic relationships in the overall network were eliminated. Fig. 5 (like Fig. 3) depicts the overall gene network with the remaining relationships, and the BIC score for the overall network was computed. Steps between Fig. 3 and Fig. 5 were repeated until the overall network with the best BIC was identified.
