# Supplementary material for: Inference of gene pathways using mixture Bayesian networks
Source: BMC Syst Biol. 2009 May 19;3:54. doi: 10.1186/1752-0509-3-54 (PMC2701418; doi:10.1186/1752-0509-3-54)
Supplement: Additional file 3 — Estimation of mixture parameters. Formulae to estimate the parameters using the Expectation-Maximization algorithm. [file 1752-0509-3-54-S3.pdf]

The Expectation-Maximization (EM) algorithm was used to estimate the mean and variance-covariance parameters of the mixture components considered for each gene sub-network. In the Expectation (E) step, the probability that each observation pertains to each mixture component, given the parameter values estimated in the Maximization step, are obtained. In the Maximization step (M), parameters are estimated as the values that maximize the restricted-log likelihood of the observed data, given the distribution of the observations over the mixture components. The equations in the Expectation and Maximization steps corresponding to the sub-network of the *jth* gene are:

Expectation step:

$$w_{jik} = P\left(m_{jk} \middle| \mathbf{x}_{ji}, \theta\right) = \frac{P\left(m_{jk} \mid \theta\right) P\left(\mathbf{x}_{ji} \middle| m_{jk}, \theta\right)}{P\left(\mathbf{x}_{ji} \mid \theta\right)}$$

Maximization step:

$$\alpha_{jk} = \frac{\sum_{i=1}^{D} w_{jik}}{D} \qquad \boldsymbol{\mu}_{jk} = \frac{\sum_{i=1}^{D} w_{jik} \mathbf{x}_{ji}}{\sum_{i=1}^{D} w_{jik}}$$

$$\boldsymbol{\Sigma}_{jk} = \frac{\sum_{i=1}^{D} w_{jik} \left(\mathbf{x}_{ji} - \boldsymbol{\mu}_{jk}\right)\left(\mathbf{x}_{ji} - \boldsymbol{\mu}_{jk}\right)^{T}}{\sum_{i=1}^{D} w_{jik}}$$

where w_{jik} denotes the evidence that the observation (gene expression) vector $\mathbf{x}_{ji}$ corresponding to the *jth* gene sub-network pertains to the *kth* multivariate Gaussian mixture component m_{jk} (k = 1 to *M*), and θ denotes all the unknown parameters across all the mixture components. Within a gene, the observations were assumed to be identically and independently distributed. This assumption can be relaxed to accommodate heterogeneity of variance (e.g. some conditions result in more variable gene expression

observations than others) and covariance between observations (e.g. gene expression observations from subsequent time points or similar treatment dosages are expected to be more correlated to each other than to observations from distant time points or more dissimilar treatment dosages). The system of EM equations was iterated until convergence.
