# Supplementary material for: Inference of gene pathways using mixture Bayesian networks
Source: BMC Syst Biol. 2009 May 19;3:54. doi: 10.1186/1752-0509-3-54 (PMC2701418; doi:10.1186/1752-0509-3-54)
Supplement: Additional file 4 — Assignment of yeast cell cycle conditions to mixture components. Distribution of yeast cell cycle treatments across the mixture components of the gene sub-network. [file 1752-0509-3-54-S4.pdf]

For the α-factor synchronization, samples were collected every seven minutes and the assignment of samples to mixture components suggests that the *CDC5* sub-network had one behavior at early sampling time-points and another behavior at later time points. Samples collected between 0 to 35 minutes after α-factor synchronization were assigned to one mixture component, and the vast majority of the samples collected from 49 to 119 minutes were assigned to the other mixture component. Only the samples corresponding to 77 and 91 minutes were assigned to the first mixture component even though the samples at the neighboring time-points were all assigned to the second mixture component. The sample corresponding to the intermediate sampling time-point of 42 minutes had a similar probability to pertain to either mixture component. Likewise, for the cdc28-synchronization samples collected every 10 minutes from 0 to 160 minutes, the five samples collected from 0 to 40 minutes were assigned to one mixture component and most of the samples collected after 40 minutes (8 out of 10 samples) were assigned to the other mixture component. These results suggest that the *CDC5* sub-network had one behavior at shortly after cdc28-synchronization and another behavior long after synchronization.

Investigation of the assignment of cdc15-synchronized samples collected every 10 minutes for 300 minutes to mixture components did not provide definitive evidence of stabilized networks across time-points with the exception of the six samples collected between 130 and 180 minutes that were all assigned to the same mixture component. The 18 samples obtained before 130 minutes and after 180 minutes had similar probabilities of pertaining to both mixture components or, when clearly assigned to a single mixture

component, no more than four consecutive samples were assigned to the same mixture component. Lastly, both Cln3 samples and both Clb2 samples were assigned to the same mixture component.
