# Supplementary material for: Inference of gene pathways using mixture Bayesian networks
Source: BMC Syst Biol. 2009 May 19;3:54. doi: 10.1186/1752-0509-3-54 (PMC2701418; doi:10.1186/1752-0509-3-54)
Supplement: Additional file 5 — Additional algorithmic extensions. Potential modifications of the proposed algorithm are discussed. [file 1752-0509-3-54-S5.pdf]

Multiple mixture model components provide flexibility to describe condition-specific gene patterns and relationships. Boutilier et al. [31] presented an algorithm to capture context-specific independencies induced by specific variable assignments, but this approach was not applied to networks. Our approach differs from Boutilier et al. [31] in that, if the network including a relationship has a higher BIC score than the network excluding the relationship, then the final network will include the relationship, and the mixture parameter estimates can be used to detect conditions in which the covariance between the genes is nearly zero. The identification of relationships between genes implemented in this study relies on the comparison of the BIC score of the full (parent-child relationship) and reduced (child alone) models. Thus, in addition to the comparison of BIC scores, other variable selection approaches (e.g. forwards selection [32], backward elimination [32], LASSO [33]) can also be used to identify the gene relationships supported by the data.

The use of a mixture of Gaussian distributions to describe the probability density function of the genes had numerous advantages including ease of interpretation of parameter estimates, identification of condition-dependent or independent gene behaviors (location or mean, dispersion or variances) and relationships (co-dispersion or co-variances), and availability of the EM algorithm to estimate mixture model parameters applied to Bayesian networks [8, 11]. The approach can be extended to consider other distributional assumptions such as mixtures of t- or Cauchy distributions. Likewise, Cobb and Shenoy [34] used a mixture truncated exponential densities to describe hybrid Bayesian networks containing discrete nodes and continuous parents, and applied this model to a waste

incinerator emission and crop price networks. Additionally, non-mixture distributions that capture the potential complexity of the probability distribution across conditions can be considered. For example, although not applied to Bayesian networks and condition-dependent gene network profiles, Purdom and Holmes [35] proposed using asymmetric Laplace distributions to describe the log-ratio of gene expression measurements. Likewise, Khondoker et al. [36] used a Cauchy distribution to account for gene expression outliers and pixel censoring, Kuznetsov [37] and Kuruoglu et al. [38] used Pareto and Pareto-tail distributions (stable distribution) to capture potentially skewed gene expression distributions, and Hoyle et al. [39] used log-Normal distributions to describe spot intensities.

The results presented in this study assume no prior knowledge of any mixture parameter (e.g. covariance between genes) or network structure (parent-child gene relationships). This approach evaluated the ability of the method to detect true relationships between genes, and precisely characterize gene co-expression profiles, regardless of the information available. Within the Bayesian framework, knowledge or belief of any unknown parameter can be incorporated through prior distributions and corresponding hyper-parameters [5]. For example, Werhli and Husmeier [40, 41] described the integration of prior knowledge to improve the reconstruction of gene regulatory networks by expressing the prior knowledge in terms of energy functions. This approach can be used to combine data from different experiments, but it can also result in higher computational demands.
